# Modeling Wildfire Incident Complexity Dynamics

**DOI:** 10.1371/journal.pone.0063297

**Published:** 2013-05-14

**Authors:** Matthew P. Thompson

**Affiliations:** Rocky Mountain Research Station, United States Forest Service, Missoula, Montana, United States of America; Universita’ del Piemonte Orientale, Italy

## Abstract

Wildfire management in the United States and elsewhere is challenged by substantial uncertainty regarding the location and timing of fire events, the socioeconomic and ecological consequences of these events, and the costs of suppression. Escalating U.S. Forest Service suppression expenditures is of particular concern at a time of fiscal austerity as swelling fire management budgets lead to decreases for non-fire programs, and as the likelihood of disruptive within-season borrowing potentially increases. Thus there is a strong interest in better understanding factors influencing suppression decisions and in turn their influence on suppression costs. As a step in that direction, this paper presents a probabilistic analysis of geographic and temporal variation in incident management team response to wildfires. The specific focus is incident complexity dynamics through time for fires managed by the U.S. Forest Service. The modeling framework is based on the recognition that large wildfire management entails recurrent decisions across time in response to changing conditions, which can be represented as a stochastic dynamic system. Daily incident complexity dynamics are modeled according to a first-order Markov chain, with containment represented as an absorbing state. A statistically significant difference in complexity dynamics between Forest Service Regions is demonstrated. Incident complexity probability transition matrices and expected times until containment are presented at national and regional levels. Results of this analysis can help improve understanding of geographic variation in incident management and associated cost structures, and can be incorporated into future analyses examining the economic efficiency of wildfire management.

## Introduction

Wildfire management in the United States and elsewhere is challenged by substantial uncertainty regarding the location and timing of fire events, the socioeconomic and ecological consequences of these events, and the costs of suppression [1]. Escalating U.S. Forest Service (Forest Service) suppression expenditures is of particular concern at a time of fiscal austerity as swelling fire management budgets lead to decreases for non-fire programs, and as the likelihood of disruptive within-season borrowing potentially increases [2]. Hence there is a strong interest in better understanding factors influencing suppression decisions and in turn their influence on suppression costs [3]–[6].

At a basic level, suppression costs are a function of the type and amount of firefighting resources assigned to an incident on a daily basis, as well as the duration of the incident. Most existing suppression cost models are statistically based however and do not explicitly consider suppression operations or firefighting resource use throughout the duration of a wildfire [7]–[11]. Thus a dynamic model of suppression operations and incident management organizations would be useful.

**Figure 1:**
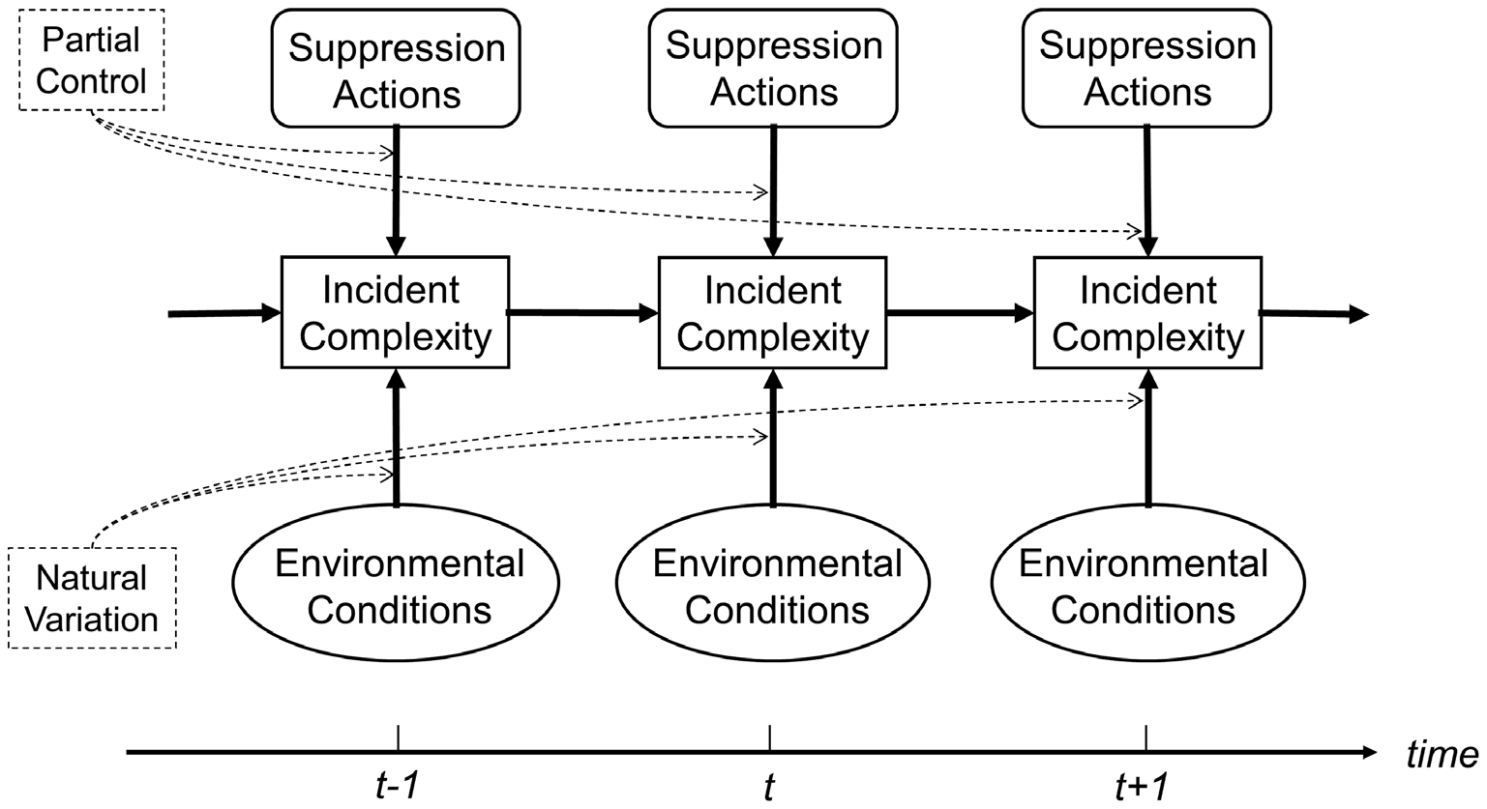
Model of the evolution of wildfire incident complexity dynamics through time. At each time step (day), suppression actions and environmental conditions jointly influence the complexity of the incident. Stochasticity in system evolution is due to partial control and natural variation. System evolution begins with incident detection and ends with containment. Adapted from [12].

The management of large wildfire incidents presents a complex and dynamic decision environment, entailing recurrent decisions across time in response to changing conditions. The state of the system at any point in time is a multidimensional composite of many attributes, which for organizational and managerial purposes is defined by the complexity of the incident (see Incident Complexity and Organizational Needs). Figure 1 conceptualizes the evolution of wildfire incident complexity through time as a stochastic, dynamic system. At each time step, suppression decisions and environmental conditions jointly influence the state of the system, but partial control (i.e., limited suppression effectiveness) and environmental variation (i.e., fire weather conditions) contribute uncertainty to system dynamics [13]–[14]. Better understanding how fire managers navigate this decision environment and how decision support information is used could ultimately lead to an improved ability to evaluate tradeoffs and to develop optimal suppression strategies [15].

Greater incident complexity is typically associated with increased demand for firefighting resource use and thus suppression costs. Incident cost structures of course will vary with incident objectives, firefighting resource needs, and incident duration, as well as local environmental factors. Nevertheless a better understanding of geographic variation in incident complexity dynamics and corresponding management needs could help better identify factors influencing suppression costs and inform potential fire management investments. Understanding geographic variation in likely incident management needs could also help identify a suite of mitigation factors to reduce risks and costs, including geographic targeting for investments in pre-fire planning and response capacity.

Previous work has examined the stochastic dynamics of environmental conditions that influence fire occurrence and behavior. Martell, for instance, modeled daily changes in the Fire Weather Index of the Canadian Forest Fire Weather Index System according to a Markov chain model [16], and Boychuk and Martell similarly modeled daily changes in fuel moisture conditions and associated fire management work load as a Markov chain [17]. More recently, Chow and Regan used a Markov chain approach to model daily changes in the Burning Index from the U.S. National Fire Danger Rating System [18]. In the fire behavior realm, Boychuk et al. [19] and Krougly et al. [20] have used Markov chains to model fire spread across gridded landscapes (see also [21]).

Here, analysis of Markov chain applications in the wildfire context is expanded to consider suppression decision making and incident complexity dynamics. That is, this work considers not only changing environmental conditions and/or fire growth, but also their relation to wildfire management, recognizing that incident complexity stems from a coupled human-natural system. This model is largely consistent with the event-frame model of incident analysis proposed by MacGregor and González-Cabán, where management of an incident evolves in discrete steps in response to changing environmental conditions [22].

**Figure 2:**
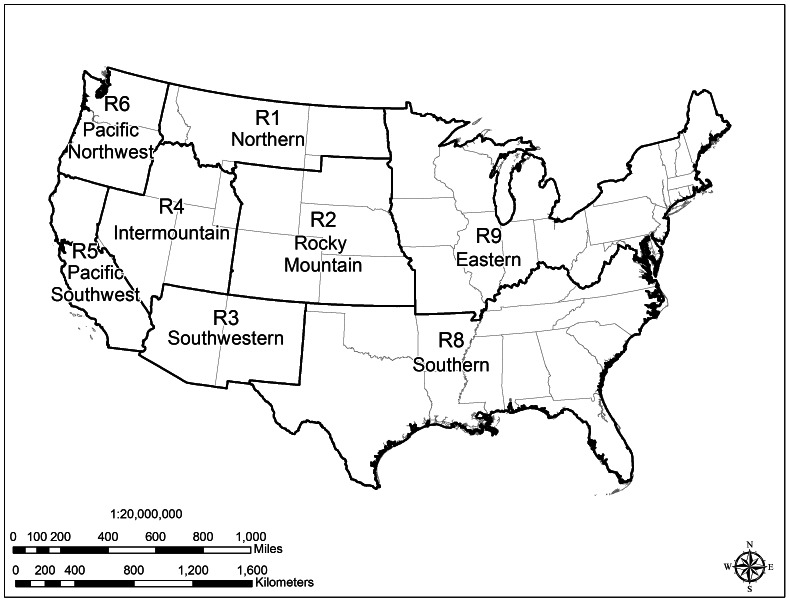
Forest Service Region numbers and names for the continental United States.

In the sections that follow, first a background on incident complexity and incident management organization is provided. Next the Markov chain model is presented as well as the two primary analytical metrics – state transition probabilities and expected times until containment. Data and results are presented for national and regional analysis scales, demonstrating significant differences in incident complexity dynamics and incident durations across Forest Service Regions (see Figure 2). Lastly implications of this work and future research directions are discussed.

### Incident Complexity and Organizational Needs

For escaped large wildfires managed in the United States, the Incident Command System builds organizational capacity (equipment, personnel, etc.) according to the complexity of the wildland fire event, ranging from Type 5 (least complex) to Type 1 (most complex). Wildfire management is based upon flexible and scalable response organizations, built on a tiered system at local, regional (Geographic Area), and national levels. Type 1 and 2 Incident Management Teams (IMTs) are national and regional resources, respectively, whereas Types 3–5 are more local. All wildland fires have a qualified Incident Commander (IC), who is responsible for maintaining command and control of the incident management organization and deploying tactical and operational decisions to achieve incident objectives. Agency administrators set incident-level strategic objectives, delegate authority to the IC, and help assess organizational needs. Continual reassessment of the complexity of the incident ensures the appropriate command organization is in place. Regulations limiting the length of time a given IC or IMT can remain on an incident lead to rotations, but not necessarily to changes in the qualifications of the next IC. Formalized incident complexity analysis templates facilitate the assessment of the current command organization, and include primary factors such as fire behavior, firefighting resources committed, firefighter safety, natural and developed resources threatened, ownership and jurisdiction, and external influences (sociopolitical concerns, etc.).

The National Wildfire Coordinating Group recently adopted an Organizational Needs Assessment (ONA) as a replacement for the Type 1–3 incident complexity analysis template. The aim of the ONA is to assist in situational assessment of complex incidents and in evaluation of objectives, risks, and management considerations to determine the appropriate management organization. The ONA framework is hierarchical and includes multiple attributes, considering qualitative descriptions (low, moderate, high) of three primary attributes: Relative Risk, Implementation Difficulty, and Decision Concerns. Assessed values for each attribute are incorporated into a needs assessment chart to identify the appropriate IMT. Assessment of Relative Risk entails consideration of three attributes, Values, Hazard, and Probability, which themselves are similarly a qualitative composite of multiple attributes. Assessment of Implementation Difficulty provides an opportunity to identify local information regarding historic fire duration, special needs and concerns, and potential tactical responses. Decision Concerns indicate the difficulty and complexity of the fire, in particular with respect to issues that may influence and affect the decision space and range of options. Additional information on interagency standards for wildfire incident management can be found in the National Interagency Fire Center’s “Red Book” [23].

The Wildland Fire Decision Support System (WFDSS), used by the Forest Service and other federal land management agencies for incident assessment and decision documentation, contains functionality to analyze incident complexity and organizational needs [24]. Spatial decision support functionality within WFDSS, including fire behavior modeling and quantification of exposure levels for values-at-risk, can assist with situational assessment, in particular Relative Risk [25]. Per WFDSS reporting protocols, decisions are documented within the system when incident complexity changes.

## Methods

### A Markov Model of Incident Complexity Dynamics

**Figure 3:**
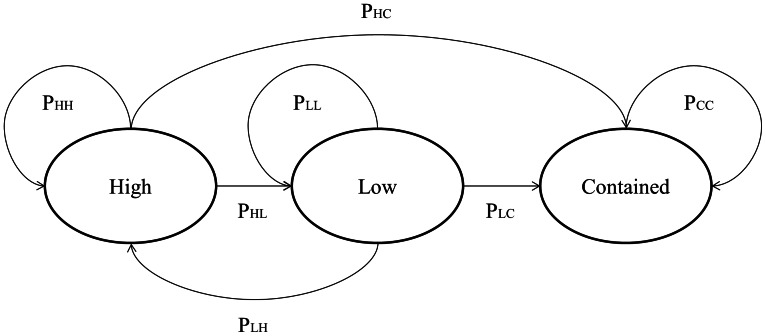
Conceptual representation of Markov Chain model of incident complexity dynamics. Here only three states – high complexity, low complexity, and incident contained – are used to simplify presentation of the system. The contained state is presented as an absorbing state (i.e., P_CC_ = 1).

Figure 3 presents a conceptual representation of a first-order Markov chain model of incident complexity dynamics. To simplify the presentation here only three states are used (H = high, L = low, and C = contained). In this model, at each time step, incident complexity probabilistically evolves. *P_LH_* corresponds to the probability of transitioning from a low complexity level today to a high complexity level tomorrow, and so forth. Once an incident is reported as contained, it is assumed to remain that way, and incident evolution ends (i.e., ‘incident contained’ is an absorbing state).

The entire system can be characterized by the state transition probability matrix, **P**, a square matrix where each element *P_ij_* of the matrix is located in the *i* ^th^ row and the *j* ^th^ column and corresponds to the probability of the system being in state *j* tomorrow given it is in state *i* today.

Modeling daily changes in incident complexity requires first identifying the order of the Markov chain – here a first-order chain is used – and then quantifying state transition probabilities. The state transition probability matrix therefore is a 6×6 matrix, corresponding to Types 1–5 and the additional absorbing state ‘contained’.

The state transition probability matrix, **P**, is calculated as follows. Let *n_ij_* be the number of times a transition from state *i* to state *j* is observed. Each *P_ij_* entry is calculated as shown in Equation 1. A derived variable of interest is the cumulative probability of incident complexity increasing (*CPI*) to Type 1 or Type 2, conditional on an incident currently being in a lower complexity state. Higher *CPI* values are indicative of incident escalations requiring non-local resources (Equation 2).
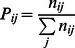
(1)

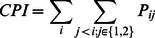
(2)


Recognizing that **P** is an absorbing chain allows an alternate formulation as shown in Equation 3, where **I** is an identity matrix, **R** a rectangular sub-matrix with transition probabilities from transient (i.e., non-absorbing) states to absorbing states, and **Q** is a square (5×5) sub-matrix giving transition probabilities among transient states. The fundamental matrix, **N**, can be calculated as shown in Equation 4, where each entry *N_ij_* is the expected number of times the chain is in transient state *j* given it started in transient state *i*. The expected time until containment given the chain started in state *i*, *E(T_i_)*, is calculated as shown in Equation 5.
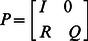
(3)

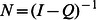
(4)

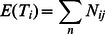
(5)


To analyze the results I followed statistical inference procedures outlined in [16] and [26]. Specifically I tested two hypotheses. In the first test the null hypothesis is that the Markov chain is of order 0, against the alternative hypothesis that the Markov chain is of order 1. This test is performed for both national and regional results. The test statistic has a χ^2^ distribution with *(m-1)^2^* degrees of freedom, where *m* is the number of states. In the second test the null hypothesis is that all 8 regional Markov chains are samples from the same Markov chain, i.e., that regional incident complexity processes are identical. This test statistic has a limiting χ^2^ distribution with *m^r^(m-1)(s-1)* degrees of freedom, where *m* is the number of states, *r* is the order of the Markov chain, and *s* is the number of samples, or regions.

### Incident Complexity Analysis

Data on incident complexity transitions come from Incident Status Summary (ICS-209) reports and span fiscal years 2002 through 2011 (the U.S. federal government’s fiscal year begins on October 1 and ends September 30). Selection criteria included only Forest Service fires lasting longer than one day, and excluded incidents labeled as wildland fire use events, since over that time frame policy changes removed fire use as a distinct type of incident. This initial dataset contained 1,422 incidents. With these reports I compiled daily incident complexity dynamics, which I broke down at the national scale and according to Forest Service Region. The Alaska Region, R10, is excluded due to too few observations.

The ICS-209 is used for reporting information on large wildfires and any other significant events on lands under federal protection or federal ownership [27]. Information is entered at the incident or dispatch level, and is used by agency managers at broader geographic planning scales for decisions related to incident prioritization and allocation of firefighting resources. ICS-209 reports are accessible online through the National Fire and Aviation Management Web Applications site (FAMWEB).

Requirements for initial reporting and the frequency of updates vary with incident-specific factors (size, management strategy, incident complexity and organizational needs), as well as with the policies of the responsible agency and the relevant interagency Geographic Coordinating Area. The minimum national requirement is submission of an ICS-209 for any fire managed under a full suppression strategy exceeding 100 acres (40 hectares) in timber, 300 acres (121 hectares) in grass and brush, or has a Type 1 or 2 IMT assigned. Significant incidents not meeting official criteria can still be reported into the system, for instance incidents threatening many values-at-risk or incidents with sensitive sociopolitical concerns. Reporting procedures require a final ICS-209 submission, typically when the incident is 100% contained or controlled, or when the remaining assigned resources are so few as to have no effect on agency resource availability.

ICS-209 forms contain 47 blocks of information, including current date and time, location, reporting status (initial, update, final), management strategy, fuels involved, values-at-risk, committed resources, estimated costs, estimated percent of contained perimeter or of management objectives completed, and, critically, IMT type. There are 14 types of IMTs, 8 of which I condensed into 5 complexity levels on the basis of the qualifications of the IC. Some of these types correspond to response organizations with a qualified IC managing the fire without being in a complete “Team” configuration, depending on resource availability and agency objectives for the incident. I excluded 6 IMT types (A–F) because they relate to either fire use or multi-agency incident management organizations. I removed all incidents where any of the reported daily IMT types were A–F. Table 1 summarizes ICS-209 information broken down by IMT types.

**Table 1:** IMT types, number of fire day observations, descriptions, and usage in the incident complexity analysis.

Incident ComplexityLevel (Modeled)	IMT Type (ICS-209)	Number of Fire Days(ICS-209)	IMT Type Description (ICS-209)
1	1	2,696	Type 1 Team
	7	3	Type 1 IC
2	2	5,242	Type 2 Team
	8	32	Type 2 IC
3	3	7,990	Type 3 Team
	4	2,888	Type 3 IC
4	5	8,315	Type 4 IC
5	6	1,803	Type 5 IC
–	A, B, F	–	Fire Use Management Team (Discontinued)
–	C, D	–	Area Command or Unified Command (Multi-agency)
–	E	–	Strategic Operational Planner (Replaces Fire Use Manager positions from Wildland Fire Use incidents)

Limitations with this dataset include missing, incomplete, or incorrect records, but many of the issues can be resolved by following ICS-209 reporting guidance and scrutinizing reporting patterns. The primary issue faced with this analysis was that for many incidents the final incident report was not submitted until well after the incident was first reported to have reached 100% containment. In part this phenomenon stems from potential errors in early reports of percent contained, but mostly this is due to untimely submission of final reports, a known issue within the ICS-209 reporting system. Whereas percent contained is a required field, information on firefighting resources committed is optional and is often not provided, and thus can’t be used as a secondary check of actual incident containment date. Approximately 33% of all incidents submitted their final report on the date when percent contained reached 100%, 60% did so within 5 days of reporting 100% containment, and further nearly 75% submitted a final report within 10 days of reporting 100% containment. A small fraction of incidents (<3%) however did not submit a final report until hundreds of days after reporting 100% containment. To be consistent across all fires, consistent with final reporting criteria, and to avoid overestimating incident durations where the final reports were filed late, I stopped counting daily transitions after the first reported instance of 100% containment, and added one transition into the absorbing containment state. In other cases, where the final report was submitted prior to 100% containment being reported, I added one daily observation reaching the containment state at the last reported entry. Secondly, another data issue related to final fire sizes that were either missing or hadn’t been updated since initial entries. For purposes of average area burned calculations I only included fire sizes greater than 100 acres (40 hectares), based in part on national minimum reporting requirements and to be consistent with the general use of ICS-209 forms for large wildfires.

## Results

Table 2 presents summary statistics on the set of fires analyzed. After filtering, this dataset included a total of 1,339 incidents, over 30,308 total fire days, for an average of 23 days per incident until containment. Regional variation in average time until containment was large, with Region 1 having the longest average time until containment (46 days), and Region 8 the shortest (9 days). Regional variation in the number of incidents is also evident, with Region 2 (80 fires) and Region 9 (35 fires) having particularly few incidents over the analysis period. Region 5 has the highest fire load (226 fires) but relatively short average time until containment (12 days). Mean fire sizes were largest in Region 3 (16,091 acres; 6,512 hectares) and smallest in Region 8 (1,469 acres; 594 hectares).

**Table 2:** Summary statistics on fires obtained from ICS-209 data, sorted according to Forest Service Region, including the proportion of active days (P(j)) in which a given incident was a given complexity level j (exclusive of containment).

	National	R1	R2	R3	R4	R5	R6	R8	R9
**Number of Fires**	1,339	217	80	232	200	226	147	202	35
**Total Daily Observations**	30,308	9,977	1,852	4,037	5,487	2,730	3,982	1,809	434
**Average Time Until Containment (days)**	23	46	23	17	27	12	27	9	12
**Average Size (acres)**	9,934	9,503	7,492	16,091	11,160	12,880	11,964	1,469	3,255
**P(1)**	0.09	0.04	0.06	0.09	0.07	0.32	0.15	0.04	0.10
**P(2)**	0.18	0.13	0.17	0.19	0.14	0.34	0.27	0.13	0.21
**P(3)**	0.38	0.35	0.47	0.35	0.36	0.28	0.47	0.50	0.25
**P(4)**	0.29	0.40	0.21	0.36	0.30	0.05	0.09	0.30	0.32
**P(5)**	0.06	0.07	0.09	0.01	0.14	0.00	0.02	0.03	0.12

Perhaps more informative are regional differences in the proportion of time that active incidents are of a given complexity type. Nationally only 27% of fire days are of Type 1 or 2, with the remainder of Type 3–5 and thus likely managed by local firefighting resources. Most regions have comparable proportions of Type 1 and 2 fire days. Regions 5 and 6 by contrast have large proportions of fire days with Type 1 and 2 incident complexity levels, 66% and 42%, respectively.

Table 3 presents the maximum likelihood estimates for daily transition probabilities for incident complexity at the national level, and Table 4 presents identical information organized according to Forest Service Region. Table 5 presents the results of the likelihood ratio statistical tests for null hypothesis that chain is of order 0 against alternative hypothesis that chain is of order 1 (hypothesis 1). Results for the national and regional first-order Markov chains are significant at the p<0.005 level. Similar statistical results apply for the second hypothesis, rejecting the null hypothesis that all 8 regional Markov chains are identical processes (test statistics is 1,127.72; 210 degrees of freedom; p<0.005).

**Table 3:** Daily transition probabilities between incident complexity levels, presented at the national scale.

National						
	1	2	3	4	5	C
**1**	0.93	0.02	0.01	0.00	0.00	0.03
**2**	0.02	0.89	0.02	0.00	0.00	0.06
**3**	0.01	0.03	0.90	0.01	0.00	0.05
**4**	0.00	0.00	0.01	0.95	0.00	0.03
**5**	0.00	0.00	0.00	0.01	0.96	0.04
**C**	0.00	0.00	0.00	0.00	0.00	1.00

**Table 4:** Daily transition probabilities between incident complexity levels, sorted according to Forest Service Region.

R1							R5						
	1	2	3	4	5	C		1	2	3	4	5	C
**1**	0.94	0.03	0.02	0.00	0.00	0.01	**1**	0.94	0.02	0.01	0.00	0.00	0.03
**2**	0.01	0.94	0.02	0.00	0.00	0.03	**2**	0.03	0.83	0.02	0.00	0.00	0.12
**3**	0.00	0.01	0.95	0.01	0.00	0.02	**3**	0.02	0.12	0.75	0.00	0.00	0.12
**4**	0.00	0.00	0.00	0.98	0.00	0.02	**4**	0.00	0.00	0.01	0.92	0.00	0.07
**5**	0.00	0.00	0.00	0.00	0.97	0.03	**5**	0.00	0.00	0.00	0.00	0.00	1.00
**C**	0.00	0.00	0.00	0.00	0.00	1.00	**C**	0.00	0.00	0.00	0.00	0.00	1.00
**R2**							**R6**						
	**1**	**2**	**3**	**4**	**5**	**C**		**1**	**2**	**3**	**4**	**5**	**C**
**1**	0.90	0.01	0.01	0.00	0.00	0.08	**1**	0.94	0.02	0.02	0.00	0.00	0.02
**2**	0.01	0.89	0.04	0.00	0.00	0.06	**2**	0.02	0.89	0.03	0.00	0.00	0.06
**3**	0.01	0.03	0.91	0.01	0.00	0.04	**3**	0.00	0.04	0.92	0.00	0.00	0.04
**4**	0.00	0.00	0.01	0.96	0.00	0.03	**4**	0.00	0.00	0.01	0.96	0.00	0.03
**5**	0.00	0.00	0.00	0.00	0.96	0.04	**5**	0.00	0.00	0.00	0.01	0.97	0.01
**C**	0.00	0.00	0.00	0.00	0.00	1.00	**C**	0.00	0.00	0.00	0.00	0.00	1.00
**R3**							**R8**						
	**1**	**2**	**3**	**4**	**5**	**C**		**1**	**2**	**3**	**4**	**5**	**C**
**1**	0.89	0.03	0.02	0.00	0.00	0.06	**1**	0.86	0.00	0.06	0.00	0.00	0.08
**2**	0.01	0.87	0.03	0.01	0.00	0.08	**2**	0.00	0.92	0.02	0.01	0.00	0.05
**3**	0.01	0.04	0.86	0.02	0.00	0.07	**3**	0.01	0.01	0.82	0.02	0.00	0.14
**4**	0.00	0.00	0.01	0.94	0.00	0.04	**4**	0.00	0.00	0.03	0.82	0.01	0.14
**5**	0.00	0.00	0.00	0.00	0.88	0.12	**5**	0.00	0.00	0.00	0.00	0.89	0.11
**C**	0.00	0.00	0.00	0.00	0.00	1.00	**C**	0.00	0.00	0.00	0.00	0.00	1.00
**R4**							**R9**						
	**1**	**2**	**3**	**4**	**5**	**C**		**1**	**2**	**3**	**4**	**5**	**C**
**1**	0.93	0.01	0.01	0.00	0.00	0.04	**1**	0.93	0.05	0.00	0.00	0.00	0.03
**2**	0.01	0.89	0.02	0.01	0.00	0.07	**2**	0.04	0.86	0.04	0.00	0.00	0.07
**3**	0.01	0.03	0.91	0.01	0.00	0.04	**3**	0.00	0.06	0.79	0.00	0.00	0.15
**4**	0.00	0.00	0.01	0.96	0.00	0.02	**4**	0.00	0.01	0.01	0.89	0.00	0.09
**5**	0.00	0.00	0.00	0.01	0.95	0.04	**5**	0.00	0.00	0.00	0.00	0.98	0.02
**C**	0.00	0.00	0.00	0.00	0.00	1.00	**C**	0.00	0.00	0.00	0.00	0.00	1.00

**Table 5:** Results of likelihood ratio statistical tests for null hypothesis that chain is of order 0 against alternative hypothesis that chain is of order 1.

Analysis Scale	Sample size (# of Fires)	Test statistic value	Degrees of freedom
**National**	1,339	62,259.42	16
**R1**	217	21,918.95	16
**R2**	80	3,664.84	16
**R3**	232	6,560.46	16
**R4**	200	11,922.95	16
**R5**	226	3,540.52	16
**R6**	147	7,092.56	16
**R8**	202	2,144.49	16
**R9**	35	854.21	16

Within-state transition probabilities (*P_ii_*) are high, ranging from 0.89 and 0.96 at the national level. Regional within-state transition probabilities are similarly high but show greater variation, with Region 1 on the higher end and with Region 5, Region 8, and Region 9 on the lower end. Across all regions the mean within-state transition probability is 0.88. Thus daily incident complexity levels tend to remain consistent. Containment transitions probabilities (*P_iC_*; exclusive of *P_cc_*) are generally low at the national scale, and lowest in Regions 1 (0.01 to 0.03) and 6 (0.01 to 0.06). *P_iC_* values are highest in Region 9 (0.02 to 0.15), followed by Region 8 (0.05 to 0.14) and Region 5 (0.03 to 0.12). Regions with higher containment probabilities tend to have shorter average times until containment.

Table 6 presents the cumulative probabilities of incident complexity increasing (*CPI*) to Type 1 or 2. Region 5 (0.17) and Region 9 (0.10) have the highest cumulative probabilities, which is likely tied to the relatively high levels of fire days being managed as Type 1 or 2 in those regions (see Table 2). Elsewhere, regional CPI values range from just 0.03 to 0.06, and at the national scale *CPI* is 0.06. In combination with transition probability information from Tables 3 and 4, the *CPI* values lead to an interpretation that escalations to highest incident complexity levels are rare, but once an incident reaches a high complexity level it can remain there for some time.

**Table 6:** Cumulative probabilities of incident complexity increasing (CPI) to Type 1 or Type 2.

Analysis Scale	Cumulative Probability of Incident Complexity Increase
**National**	0.06
**R1**	0.03
**R2**	0.05
**R3**	0.06
**R4**	0.05
**R5**	0.17
**R6**	0.06
**R8**	0.02
**R9**	0.10

Table 7 provides the expected time until containment for each initial state *i*, *E(T_i_)*. Generally these values are consistent with average times until containment presented in Table 2. Region 1 has the longest expected time until containment, ranging from 37 to 50 days. Region 8 has the shortest expected time until containment, ranging from 7 to 15 days. Geographic variation is evident, and although there is not a strong pattern across the starting complexity level, incidents that start as Type 4 or Type 5 tend to have greater expected times until containment. Region 5 is an exception with a very short expected duration starting as Type 5, which is an artifact of a single observation of a Type 5 incident that transitioned to containment in one day.

**Table 7:** Expected time until containment for a fire that starts at a given complexity level, presented for National and Regional scales.

Initial Complexity Level	National	R1	R2	R3	R4	R5	R6	R8	R9
**1**	22	48	14	16	20	22	31	10	23
**2**	17	37	18	14	18	11	21	15	16
**3**	20	37	22	14	23	11	23	8	9
**4**	24	50	31	19	31	14	31	7	11
**5**	31	33	25	8	26	1	44	9	50

**Figure 4:**
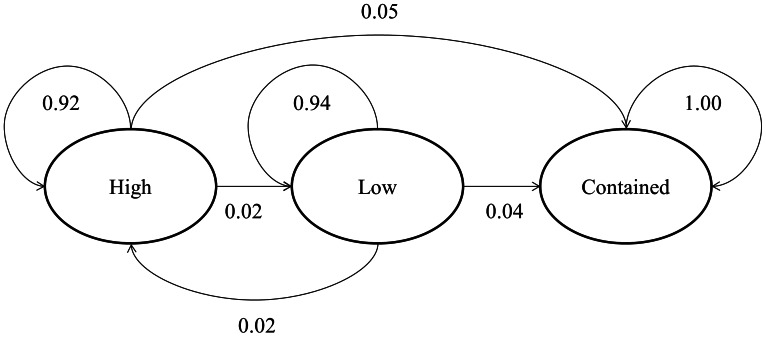
Three-state Markov Chain model of national-scale incident complexity dynamics, a simplified representation of the results presented in Table 3. Due to rounding the presented transition probabilities may not sum perfectly to 1.00.

Figure 4 presents a simplified three-state Markov model (see Figure 3), based on national-scale results and combining Types 1–2 into “high” complexity and Types 3–5 into “low” complexity. This Markov chain yields high within-state transition probabilities (0.92 to 0.94), low containment probabilities (0.04 to 0.05), and an even lower probability of incident complexity increasing from low to high (0.02). Expected time until containment for “low” complexity incidents is 22 days, and 19 days for “high” complexity incidents.

## Discussion

Incident complexity dynamics can be modeled as a Markov chain, and results of this analysis reveal significant geographic variation in transition probabilities and expected time until containment. Results reflect environmental factors influencing fire occurrence and growth, as well as socioeconomic and institutional factors influencing how fires are managed. For instance in Region 5, where the presence of dense human populations in fire-prone areas tends to lead to policies of aggressive suppression, results show a high proportion of Type 1 and 2 incidents and a short average duration.

Results are consistent with statistical cost models that find statistically significant influence of Forest Service Region on suppression costs [11]. Regions with substantially higher proportions of Type 1 and 2 complexity levels, particularly Regions 5 and 6, also tend to have higher suppression costs. This analysis highlighting likely variation in fire duration and firefighting resource use can help move towards a more mechanistic understanding of suppression operations and costs. Further, coupling this information with knowledge gained from structured post-fire processes to identify key parameters of incident decisions [22] could improve prospective assessment of fire management needs.

This analysis could also help target cost-effective fire management investments. For instance the longer expected durations for incidents that begin as Types 4 and 5 could in some cases reflect local needs for improved capacity, and preparedness investments could help reduce the frequency of fires growing to overwhelm local resources. Geographic variation in incident complexity could also inform prepositioning strategies for national resources like large airtankers and enhance initial attack effectiveness. Where wildfire risks to human communities are thought to be driving suppression response, additional analyses can consider designing protective fuel treatment strategies [28]–[30] and can examine how the arrangement and location of homes contributes to elevated risks for certain communities [31]. Results of national scale wildfire risk assessments [32] could be incorporated to project future areas of concern for high suppression costs or high levels of firefighting resource demand.

Issues with the ICS-209 reporting system are acknowledged, both in terms of gaps and potential errors with the data itself, as well as how data were analyzed. Time until containment is effectively used as a proxy for incident duration, given uncertainties associated with the submission date of the final ICS-209 report. The criterion to consider an incident contained at the first report of 100% containment could in some cases lead to underestimating actual time until containment, and it is recognized that firefighting resources and incident management teams may remain mobilized for days or longer after full containment or control is achieved. It is beyond the scope of this analysis to perform a thorough fire-by-fire evaluation to identify potential instances of or causes for a delayed final ICS-209 submission. It will likely prove useful to couple ICS-209 information with information from other databases, in particular financial accounting systems that track firefighting resources, to better identify when an incident is both reported as fully contained and has little to no firefighting resources assigned. Questions related to handling time until containment should not measurably affect modeled transition probabilities, however, and the range of modeled expected times until containment (Table 7) are consistent with observed containment times presented in Table 2. The nuances, intricacies, and contingencies of incident management are difficult to fully capture, especially working from an operational dataset, but nevertheless the modeling framework and results do nicely capture the underlying dynamics of incident complexity evolution through time.

One potential realm of improvement is the representation of the decision space. In the current model suppression decisions are captured only implicitly in the evolution of incident complexity. An obvious direction is to move towards a Markov decision process that can explicitly capture the range of decisions made by fire managers, and in turn their influence on factors affecting the evolution of incident complexity and fire outcomes. This could present a novel stochastic dynamic optimization approach for management of escaped large wildfires [33]. Management actions could be defined in terms of the addition or demobilization of firefighting resources.

Some straightforward modeling extensions and analyses are more immediately apparent. Examining seasonality trends by geographic region could identify time periods of high synchronous demand for national firefighting resources. States could be characterized with additional information such as contemporaneous regional and national fire loads, to see if incident complexity dynamics change with firefighting resource scarcity. Examining higher-order Markov chains is also possible, but comes with tradeoffs in terms of an increased state space and a reduced sample size as short-duration incidents would be excluded. Further, building off the decision process idea introduced above, it seems likely that decisions to escalate or de-escalate incident complexity would be based more on current fire conditions and available resources than on the temporal duration of the existing incident complexity level.

Longer term work could seek to better account for additional sources of uncertainty in the system presented in Figure 1. First, additional uncertainty stems from partial observability of the true system state, a commonality among natural resource management problems [34]. Partial observability could exist, for instance, in terms of uncertainty regarding the current fire perimeter, fire behavior, and the exact location of threatened natural resources such as nesting sites. Second, additional uncertainty stems from structural uncertainty regarding the model of state transitions. Structural uncertainty is present for instance in terms of how we understand and model fire spread and fire containment [35], [14].

Continued analysis of the management of escaped large wildland fires should help improve models of suppression operations and suppression decision making, and ultimately improve our ability to forecast and manage suppression costs. An improved ability to forecast fire management budgets and firefighting resource demands should prove increasingly useful, particularly in the face of a changing climate and expanding human development into fire-prone areas.
